# Multiple blood pathogen infections in apparently healthy sheltered dogs in southern Thailand

**DOI:** 10.1080/23144599.2022.2111514

**Published:** 2022-08-24

**Authors:** Narin Sontigun, Worakan Boonhoh, Punpichaya Fungwithaya, Tuempong Wongtawan

**Affiliations:** a Akkhraratchakumari Veterinary College, Walailak University, Nakhon Si Thammarat, Thailand; b Centre for One Health, Walailak University, Nakhon Si Thammarat, Thailand; c Centre of Excellence Research for Melioidosis and Other Microorganism, Walailak University, Nakhon Si Thammarat, Thailand

**Keywords:** Blood pathogen, dog, shelter, Thailand

## Abstract

In developing countries such as Thailand, free-ranging dogs are frequently involved in road accidents and contribute to the cost of public healthcare. Shelters play a vital role in communities because they help to control the population of unwanted and free-ranging dogs. This study aimed to investigate blood pathogen infection in sheltered dogs, as it is one of the factors contributing to animal welfare. Blood samples were randomly collected from 141 dogs from the largest shelter (approximately 400–500 dogs in total) in southern Thailand. Blood pathogens were detected using both PCR and light microscopy. Four blood pathogens were identified: *Anaplasma platys, Ehrlichia canis, Babesia canis vogeli*, and *Hepatozoon canis*. No trypanosomes were detected. The incidence of blood parasite infection was 56.7% (80/141) by PCR, and 28.4% (40/141) by microscopy. *E. canis* was the most prevalent pathogen, accounting for 46.1% (65/141) of the cases, while multiple infections accounted for 22% (31/141) of the cases. A triple infection with *E. canis, A. platys*, and *B. canis vogeli* was observed in 5.7% (8/141) of the cases. Although PCR is far more sensitive than microscopy, it appears to have equivalent specificity. In conclusion, this study reported a high occurrence of blood pathogen infections in clinically healthy sheltered dogs. Many of them were infected with multiple pathogens and may have been infected before entering the shelter. These findings suggest that a blood test is necessary to screen dogs prior to their admission to the shelter to prevent disease transmission and enhance animal welfare.

## 1. Introduction

According to estimates, Thailand has approximately 13 million dogs, the majority of which are owned, while 13% are stray dogs, 5% reside in temples, referred to as temple dogs [1]. Many owners do not keep dogs in their homes, but feed and allow them to roam freely in the community (also referred to as free-ranging dogs) [2,3]. It has been speculated that the percentage of owned free-ranging dogs in Thailand may exceed 50% of all owned dogs (approximately 4 million) [4]. Apart from the dangers of road accidents and biting, free-ranging and stray dogs carry many diseases that can spread to other animals and humans. These diseases include rabies, leptospirosis, and parasitic diseases [5–7].

Shelters are critical to communities as they help to control the population of unwanted, free-ranging, or strayed dogs and find new homes [8]. Approximately 30 dog shelters in Thailand have appeared on the internet, all of which are no-kill shelters. Each shelter house has between 100 and 5000 dogs, but the average is approximately 500 (personal communication). With a no-kill policy, an excessive number of dogs live in shelters, making it difficult to manage their health and well-being, and they could become a source of disease transmission to other dogs, animals, and humans [8,9]. However, there have been a limited number of reports on the health, disease, and welfare of sheltered dogs, mainly in developing nations. In Thailand, one study discovered a blood pathogen infection in shelter dogs, but this study area was limited to the northern and central areas [10].

Blood pathogens are disease-causing agents in domestic dogs worldwide and have a negative impact on health and welfare [11–15]. Four species of blood pathogens are commonly reported in Thailand, including *B. canis, H. canis, E. canis*, and *A. platys* [13,16], all of which share a common vector, the brown dog tick (*Rhipicephalus sanguineus*), the most common tick species found in dogs in Thailand and the world [17,18].

In Thailand, investigations of blood pathogens are often conducted on stray dogs, with an occurrence of between 35% and 76% [7,19–24], and on owned dogs who visit or stay in hospitals, with an occurrence of between 14% and 57% [13,16,18,25–28]. However, only one study examined the prevalence of blood pathogens in two sheltered dogs in northern and central Thailand, and found that the infection occurred at a rate of approximately 25% [10]. Due to insufficient data from sheltered dogs, particularly in southern Thailand, the objectives of this study were to investigate the occurrence of blood pathogen infections in sheltered dogs in this region, and to compare the diagnostic methods between microscopy and PCR.

## 2. Materials and methods

### 2.1. The shelter

This is the largest shelter in southern Thailand, with approximately 400–500 dogs from all over the region. The 2,000 m^2^ open-air style shelter was divided into four stables. Each stable consists of concrete with a roof covering 50% of the space, and the remaining area is the ground area with few trees. The floor and soil were treated monthly with a diluted insecticide, Bayticol (Bayer, Leverkusen, Germany), to control ticks and fleas in the environment.

All dogs in the shelter were mixed breeds, mainly native Thai dogs. Most dogs in the shelter were females (approximately 70%). The dogs were provided commercial food and water ad libitum. Dogs were annually vaccinated with multiple vaccines (rabies, distemper, adenovirus type 2, parainfluenza, parvovirus, leptospirosis, and coronavirus), monthly injected with ivermectin (to prevent internal and external parasites), and showered weekly.

### 2.2. Animal sampling

Blood samples (3 ml each) were collected randomly from 141 dogs (approximately 30% of the population) between February and May 2021. The estimated sample size for studying prevalence was calculated using EPITOOLS online software (https://epitools.ausvet.com.au) with the estimated proportion set to 0.1, the desired precision of the estimate set to 0.05, with a confidence level set to 0.95, and the sample size was calculated to be between 140 and 150.

The inclusion criteria were dogs without clinical signs of any disease from external appearance, a body score of 3/5, weight of approximately 10 kg, and dog aged 2–9 years old. The exclusion criteria were highly aggressive and anorexic dogs. The samples were mostly from female dogs (n = 104), which were the main population in this shelter. All dogs were neutralized. Blood was collected from the cephalic vein by using a syringe with a 22-gauge needle (NIPRO, Thailand). One or two ticks (*Rhipicephalus sanguineus*) were found in five dogs.

### 2.3. Detection of blood pathogens

Five species of blood pathogens (*B. canis vogeli, E. canis, H. canis, A. platys*, and *Trypanosoma* spp.) were investigated using conventional polymerase chain reaction (PCR) and microscopy.

For microscopy, a drop of blood was smeared onto a glass slide and air-dried at room temperature. Slides were fixed with methanol and stained with 10% Giemsa. Next, the slides were washed with water and air-dried before being viewed under a light microscope at 1000 × magnification (Olympus, Tokyo, Japan).

For PCR, DNA was extracted from 200 μl of blood using an E.Z.N.A.® Blood DNA Kit (Omega Bio-Tek, Norcross, GA, USA) according to the manufacturer’s instructions. The concentration of extracted DNA was measured using a Nano-Drop™ spectrophotometer (ThermoFisher Scientific, MA, USA), and then stored at −20°C until further analysis. The PCR reaction contained 6.25 µl DreamTaq Green Master Mix (2x) (Thermo Scientific, Vilnius, Lithuania), 1–2 µl DNA template (100–200 ng/uL), 0.5 µl primer (0.4 µM) (Table 1), and nuclease-free water to a final volume of 12.5 µl. PCRs were performed using a Mastercycler Pro S machine (Eppendorf AG, Hamburg, Germany). The primers used are listed in Table 1. The cycling conditions consisted of an initial denaturation step at 95°C for 3 min, followed by 35 cycles of denaturation at 95°C for 30s, annealing at 54°C (for *B. canis vogeli, E. canis*, and *H. canis*) or 58°C (for *A. platys* and *Trypanosoma* spp.) for 30s, extension at 72°C for 1 min, and a final extension at 72°C for 5 min.

**Table 1.** Primer sequences for detection of blood pathogens in dogs.

Pathogen	Gene	Oligonucleotide sequence (5´ to 3´)	Product size (bp)	Reference
*B. canis vogeli*	18S rRNA	GTGAACCTTATCACTTAAAGG CAACTCCTCCACGCAATCG	~600	[29]
*E. canis*	virB9	CCATAAGCATAGCTGATAACCCTGTTACAA TGGATAATAAAACCGTACTATGTATGCTAG	380	[30]
*H. canis*	18S rRNA	CCTGGCTATACATGAGCAAAATCTCAACTT CCAACTGTCCCTATCAATCATTAAAGC	737	[30]
*A. platys*	GroeL	TAGCTAAGGAAGCGTAGTCCGA AATAGCCGCAGCGAGCGGTTCC	275	[31]
*Trypanosoma* spp.	ITS1	CCGGAAGTTCACCGATATTG TGCTGCGTTCTTCAACGAA	250–700	[32]

For each assay, the genomic DNA of known blood pathogens was used as a positive control, whereas nuclease-free water was used as a negative control. PCR products were visualized on a 1.5% agarose gel in 1X TAE buffer and stained with SERVA DNA Stain G (SERVA, Heidelberg, Germany) under UV light using the ChemiDoc^TM^ Imaging System (Bio-Rad, CA, USA). The PCR products were confirmed by DNA sequencing (Novogene, Singapore).

### 2.4. Sensitivity and specificity

In this study, PCR was used as the gold standard for detecting blood parasites, similar to previous studies [33–35]. The sensitivity of microscopy was calculated as the percentage of positive cases by microscopy from the total PCR-positive cases (number of positive microscopy/numbers of positive PCR × 100). The specificity of microscopy was calculated as the percentage of negative cases by microscopy from the total PCR-negative cases (number of negative microscopy/numbers of negative PCR × 100).

### 2.5. Statistical analysis

Fisher’s exact test using GraphPad software (https://www.graphpad.com) was used to analyse the difference between pathogen occurrence and the difference between the results obtained by laboratory methods (microscopic diagnosis and PCR). The significance level was set at *P* < 0.05.

### 2.6. Ethical statement

This project was approved by the Institutional Animal Care and Use Committee of Walailak University (ID: 63–036).

## 3. Results

### 3.1. The overall prevalence of blood pathogen infections

**Figure 1.**
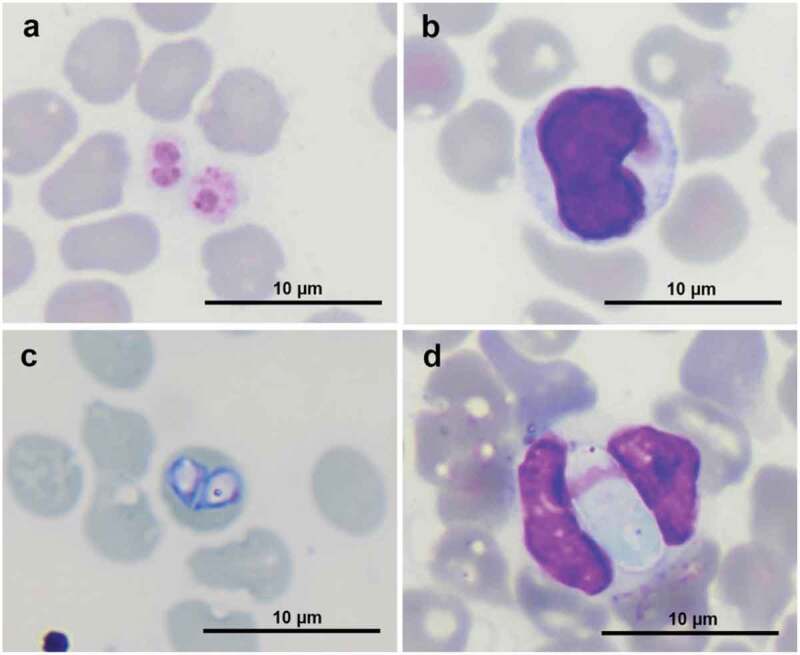
Blood pathogens in stained blood smear. (a) *A. platys* in platelets, (b) *E. canis* in a monocyte, (c) *B. canis* in a red blood cell, and (d) *H. canis* in a neutrophil at 1000 × magnification.

From 141 dogs, the PCR was able to identify 80 infected dogs (56.7%), while 40 infected dogs (28.4%) were identified by microscopy. Four blood pathogens were identified, including *A. platys, E. canis, B. canis vogeli*, and *H. canis*; however, trypanosomes were not detected (Figure 1). The overall occurrence of each blood pathogen is shown in Table 2. Using PCR, *E. canis* (46.1%) was the most predominant pathogen, and its occurrence was statistically different (*P* < 0.05) from that of other pathogens. Although *A. platys* (17.7%) was the most prevalent pathogen observed using microscopy, its occurrence was statistically different (*P* < 0.01) from other detected pathogens using the same approach. Among the four pathogens, only *E. canis* and *B. canis vogeli* were detected more frequently by PCR than by microscopy (*P* < 0.05).

**Table 2.** The occurrence of each blood pathogen in 141 dogs.

Pathogens	Number of positive samples (%)	
	By PCR	By Microscopy
*A. platys*	23 (16.3%)	25 (17.7%)
*E. canis*	65 (46.1%)	8 (5.7%)
*B. canis vogeli*	28 (19.9%)	6 (4.3%)
*H. canis*	3 (2.1%)	3 (2.1%)

### 3.2. Single and multiple infections

The occurrence of single and multiple blood pathogen infections is shown in Table 3. A single pathogen infection was detected in 34.8% of cases using PCR, and it was more frequent than that detected by microscopy (27.0%; *P* < 0.05). Double-pathogen infections were diagnosed, with only PCR at 16.3%, which was significantly higher (*P* < 0.01) than that by microscopy (1.4%). Only PCR was able to detect triple pathogen infection, with an occurrence rate of 5.7%. Overall, multiple infections were significantly detected (*P* < 0.01) by PCR (22.0%) rather than by microscopy (1.4%).

**Table 3.** The occurrence of single and multiple infections in 141 dogs.

Type of infection	Blood pathogen species	Number of positive dogs (%)	
		PCR	Microscopy
Single infection	*A. platys*	5 (3.6%)	24 (17.0%)
	*E. canis*	35 (24.8%)	6 (4.3%)
	*B. canis vogeli*	7 (5.0%)	6 (4.3%)
	*H. canis*	2 (1.4%)	2 (1.4%)
	Total single infection	49 (34.8%)	38 (27.0%)
Double infection	*A. platys + E. canis*	9 (6.4%)	1 (0.7%)
	*A. platys + B. canis vogeli*	1 (0.7%)	0 (0%)
	*A. platys + H. canis*	0	0
	*E. canis+ B. canis. vogeli*	12 (8.5%)	0 (0%)
	*E. canis+ H. canis*	1 (0.7%)	1 (0.7%)
	Total double infection	23 (16.3%)	2 (1.4%)
Triple infection	*A. platys + E. canis+ B. canis vogeli*	8 (5.7%)	0 (0%)
Multiple infection	(double and triple infections)	31 (22.0%)	2 (1.4%)

By PCR, the predominant single pathogen infection was *E. canis* (24.8%), whereas by microscopy, it was *A. platys* (17.0%). Co-infection with *E. canis* and *B. canis vogeli* (8.5%) was the main double pathogen infection identified by PCR. However, microscopy revealed that only two dogs were infected with either *A. platys* or *E. canis* (0.7%) or *E. canis* and *H. canis* (0.7%). The triple pathogen infection was identified as *A. platys, E. canis*, and *B. canis vogeli*.

### 3.3. Sensitivity and specificity of microscopy technique comparatively related to PCR

The sensitivity and specificity of microscopic examination against the PCR test are shown in Table 4. Microscopy demonstrated high specificity (> 83%), particularly for *H. canis* (100%); however, the sensitivity was relatively low, specifically for *E. canis* (16.9%), with the exception of *H. canis*, which demonstrated a maximum sensitivity of 100%, but with a low number of positive cases (n = 3). In general, the sensitivity and specificity of microscopy were significantly lower than those of PCR (*P* < 0.05).

**Table 4.** The sensitivity and specificity of microscopic examination relative to PCR test.

	A. platys	E. canis	B. canis	H. canis
Sensitivity (Microscopy/PCR positive)	34.8% (8/23)	16.9% (11/65)	21.4% (6/28)	100% (3/3)
Specificity (Microscopy/PCR negative)	83.1% (98/118)	96.1% (73/76)	98.2% (111/113)	100% (138/138)

For microscopy, the sensitivity of detecting *H. canis* was significantly higher than *E. canis* (*P* = 0.018) but was not significantly different from *A. platys* and *B. canis*. The specificity for detecting *H. canis* was statistically different from *E. canis* (*P* < 0.05) and *A. platys* (*P* < 0.0001), but not *B. canis* (*P* > 0.05).

## 4. Discussion

This study revealed multiple blood pathogen infections in sheltered dogs with no clinical signs, although very few vectors (ticks) were observed. More than half of the sheltered dog population was infected with at least one of the following blood pathogens: *E. canis, A. platys, B. canis vogeli*, or *H. canis.*

*E. canis* may be the most prevalent pathogen in Thailand, as shown in our study and previous studies, including those in the northeastern region (Maha Sarakham, Amnat Charoen, Nakhon Ratchasima, and Buriram), central region (Bangkok), western region (Kanjanaburi), and southern region (Songkhla) [7,13,16,18,21,26,27].

This is the first report of multiple blood pathogen infections in southern Thailand, where the occurrence was 22%, considerably higher than that in the northeast (2% of stray dogs) [21,25], but lower than that in the central region (36% of stray dogs) [7]. Double infection is the most common type of multiple infection in all studies in Thailand, but the combinations are diverse depending on geography and are related to the common pathogen found in those areas. The co-infection with *B. canis* and *E. canis* is dominant in the northeastern area (14%) [18]. *E. canis* and *H. canis* are commonly found (6%) in central Thailand [7], while in southern Thailand, *A. platys* and *E. canis* were found to be common co-infection (12%). Triple infections were previously found in central Thailand, with the highest incidence being the combination of *E. canis, B. canis vogeli, and H. canis* (2% [7]), whereas our study in southern Thailand showed that triple infections of *E. canis, A. platys* and *B. canis vogeli* was 7%. Quadruple blood pathogen infections (*E. canis, A. platys, B. canis vogeli*, and *H. canis*) are very rare, and are found only in central Thailand, with 0.3% of stray dogs [7]. The high prevalence of multiple infections in central Thailand may be because of the dense population of stray dogs in a small area.

Trypanosomes were not detected in dogs in this study, which is consistent with a previous study in northern and central Thailand, as well as in most neighbouring countries [36]. A decade ago, only one previous study discovered trypanosome infection in a dog travelling from Thailand to Germany [37]. However, trypanosomes have recently been detected by PCR in other species, including 2% of biting flies across Thailand [38–40] and 3% of buffalos in eastern Thailand [41]. In southern Thailand, *T. evansi* was observed in 1% of cattle by microscopy but not by PCR [42,43].

Although microscopic examination of stained blood smears is a simple and inexpensive method for diagnosing many blood pathogens, PCR-based methods offer greater sensitivity and detailed information about individual species and genetics [44,45]. This study showed that microscopy had low sensitivity for most pathogens but offered high specificity for all pathogens. A previous study recommended increasing the number of microscopic observation fields to enhance the sensitivity and specificity of results [46]. In addition, the sensitivity and specificity of blood pathogen identification by microscopy can vary based on the skill and experience of technicians [47,48].

The occurrence of *A. platys* detected by microscopy is often higher than that detected by PCR in other investigations, similar to ours [18,49]. The high occurrence by microscopy is frequently a false positive due to the detection of inclusion bodies within platelets, which may represent the morulae stage of *A. platys* [49,50]. Platelet inclusions are uncommon in dogs infected with *A. platys*, according to a previous molecular and microscopic study [50]. Moreover, various inflammatory disorders or staining errors may also cause platelet inclusion bodies, which can be misdiagnosed as *A. platys* [50,51].

In contrast to *A. platy* detection, the occurrence of *B. canis vogeli* and *E. canis* is usually higher in PCR detection than in microscopic examination. The current investigation is consistent with the previous studies [13,52,53]. While microscopy and PCR were equivalent for detecting *H. canis* in this study, other investigations have demonstrated that PCR is superior to microscopy [13,19,53]. Particularly for *E. canis*, it is rare (6%) to detect a morula in a blood smear of clinical cases, although the sensitivity can be increased by performing a buffy coat smear [54]. Although the features of *B. canis* are unique and easy to distinguish from those of other blood pathogens using microscopy, this technique has low sensitivity, particularly with low parasitaemia [55]. Samples that were previously deemed negative by microscopic analysis of blood smears were found to be positive using a more sensitive PCR approach [13,20,52].

In this study, the source of the blood pathogen infection or transmission in these sheltered dogs was not clear because these dogs came from different areas of southern Thailand and had diverse backgrounds. Most dogs were free-ranging, and some were previously owned. None of the dogs were tested for blood pathogens prior to being admitted to the shelter because screening for blood parasites before admission is an uncommon practice in Thailand, as almost all shelters operate on a limited budget and rely on free veterinary services provided by the government, volunteers, or other non-governmental organizations (personal communication). Additionally, biological vectors (ticks) and mechanical vectors (biting flies) were rarely observed in this shelter.

None of the infected dogs displayed clinical signs of infection according to the caretakers and our observations, even with multiple infections. This differs from pet dogs (pure breeds) in Thailand, which appear to be more sensitive to blood pathogens and frequently exhibit clear clinical signs (personal communication). We speculated that Thai native animals might be more resistant to blood pathogens and tropical diseases than purebreds because of their adaptation. Tolerance to blood pathogen infection has also been observed in other species, including Thai native chickens [56], and native cattle (Sontigun et al., unpublished data). Although no clinical signs were found, we observed a subclinical problem in the haematological profile of some dogs in this area, including a decreased number of erythrocytes and platelets, particularly with multiple infections (Sontigun et al., unpublished data). Several studies have also shown that dogs with co-infection (more than two blood pathogens) tend to have lower erythrocyte and platelet counts than dogs with a single infection [57–59]. For the treatment of multiple infections, our preliminary data showed that administering doxycycline at a dose of 10 mg/kg orally once a day for eight weeks could improve the haematological profile and eliminate multiple blood pathogen infections in sheltered dogs (Boonhoh et al., unpublished data).

## 5. Conclusion

This is the first report of multiple blood pathogen infections in southern Thailand, with a high occurrence of blood pathogen infection in apparently healthy sheltered dogs. Many of them were infected with multiple pathogens and may have been infected before entering the shelter. These results suggest that blood tests are necessary to screen dogs before they are admitted to the shelter to prevent disease transmission, and enhance animal welfare.
